# Hydrophobic pulses predict transmembrane helix irregularities and channel transmembrane units

**DOI:** 10.1186/1471-2105-12-135

**Published:** 2011-05-06

**Authors:** Damien Paulet, Mireille Claustres, Christophe Béroud

**Affiliations:** 1 INSERM U827, Montpellier, France; 2 Université Montpellier 1, Montpellier, France; 3 CHU Montpellier, Hôpital Arnaud de Villeneuve, Laboratoire de Génétique Moléculaire, Montpellier, France

## Abstract

**Background:**

Few high-resolution structures of integral membranes proteins are available, as crystallization of such proteins needs yet to overcome too many technical limitations. Nevertheless, prediction of their transmembrane (TM) structure by bioinformatics tools provides interesting insights on the topology of these proteins.

**Methods:**

We describe here how to extract new information from the analysis of hydrophobicity variations or hydrophobic pulses (HPulses) in the sequence of integral membrane proteins using the Hydrophobic Pulse Predictor, a new tool we developed for this purpose. To analyze the primary sequence of 70 integral membrane proteins we defined two levels of analysis: G1-HPulses for sliding windows of n = 2 to 6 and G2-HPulses for sliding windows of n = 12 to 16.

**Results:**

The G2-HPulse analysis of 541 transmembrane helices allowed the definition of the new concept of transmembrane unit (TMU) that groups together transmembrane helices and segments with potential adjacent structures. In addition, the G1-HPulse analysis identified helix irregularities that corresponded to kinks, partial helices or unannotated structural events. These irregularities could represent key dynamic elements that are alternatively activated depending on the channel status as illustrated by the crystal structures of the lactose permease in different conformations.

**Conclusions:**

Our results open a new way in the understanding of transmembrane secondary structures: hydrophobicity through hydrophobic pulses strongly impacts on such embedded structures and is not confined to define the transmembrane status of amino acids.

## Background

Integral membrane proteins (IMP) are involved in many aspects of cell physiology such as, for instance, transport of ions and solutes, cell-to-cell signaling and cell recognition. IMPs can be divided in two classes according to the characteristics (α-helix bundles or β-barrels) of their 3D structure. Helix-bundle IMPs are found in all cellular membranes, while β-barrel IMPs are only located in the outer membrane of Gram-negative bacteria, mitochondria and chloroplasts. In this paper, we will focus only on helix-bundle IMPs, as they are almost ubiquitous and represent about 25% of all open reading frames in genomes [1]. Despite their number and importance, high-resolution structures of IMPs represent only about 1% of the Protein Data Bank (PDB) entries [2] and this is mainly due to technical limitations. Therefore, bioinformatic tools play a major role in the study of IMP structures as many sequence-based algorithms provide valuable information on embedded structures. While the first predictors focused only on the detection of transmembrane (TM) regions, more recent tools are dealing with the full IMP topology, thus including both the membrane spanning and the extra/intra-cellular segments of such proteins [3].

The first principle of topology prediction relies on the average hydrophobicity of transmembrane segments (TMS). The inner cell membrane is made up mainly of aliphatic chains of phospholipids, which create a region that favors non-polar amino acids and rejects polar amino acids. To highlight regions rich in non-polar amino acids in a sequence, many propensity scales have been developed [4-8] in which each amino acid is associated with a value that can be derived from biophysical or chemical measurements (e.g., the affinity of a given amino acid for water, the free energy of insertion of an amino acid in the membrane, etc.).

**Figure 1:**
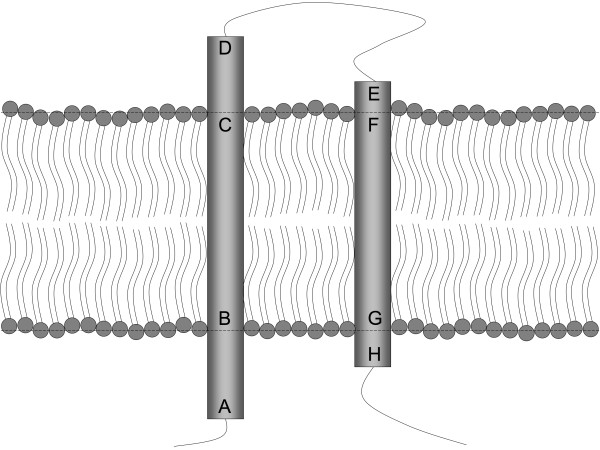
**Definition of transmembrane structures**. [A;D] is a transmembrane helix (TMH) and [B;C] represents the transmembrane segment (TMS) which is the embedded part of TMH.

More recent prediction tools rely on statistics. As TM regions share a relatively common amino acids composition, machine learning systems trained on datasets of resolved IMP should be able to detect TM segments in new proteins. Most machine learning methods rely on hidden Markov models, neural networks or support vector machine [9-11]. However, topology prediction is a far more complex operation. Whereas the first tools based on propensity scales predicted only the TMS (B-C and F-G segments in Figure 1), topology-prediction tools concentrate on the whole TM organization, i.e. the relative position of the A-D and E-H segments (the transmembrane helices, TMH) (Figure 1).

TMH formation is still a complex issue: the two-stage model by Popot and Engelman [12] and the four-step cycle by Wimley and White [13] provide interesting conceptual frameworks, but do not answer the question about how helices are formed during IMP folding. Notwithstanding, it is well recognized that thermodynamic equilibrium plays a key role as α-helices are regular structures based on hydrogen bonds, which dramatically reduce the thermodynamic cost of peptide partitioning in the membrane [14].

In this paper, we describe a method to extract new information from the analysis of hydrophobicity variations in the sequence of IMPs using the Hydrophobic Pulse Predictor (HPP), a new, freely available tool we developed for this purpose. To this end, we studied the hydrophobicity variations in the sequences of 70 non-homologous IMPs and focused on raises of hydrophobicity that we called Hydrophobic Pulses (HPulses). Our approach is different from those of different studies on variations of hydrophobicity such as hydrophobic moment. From the primary sequence of an embedded region, HPP defines the general TM organization and predicts the secondary structures. Our aim was neither to define a new hydrophobicity scale nor to predict TMS, but rather to demonstrate that hydrophobicity variations strongly impact on the secondary structures of embedded regions and that, therefore, the study of HPulses leads to a better understanding of embedded secondary structures.

## Results

In order to evaluate the impact of hydrophobicity variations (HPulses) on the structure of TM proteins, we compared the HPulses predicted using HPP to the limits of TM segments and to the extremities of 541 embedded α-helices from 70 IMPs with known 3D-structure. We defined two types of HPulses: G2-HPulses for large structural events (sliding windows of n = 12 to 16) and G1-HPulses for smaller ones (sliding windows of n = 2 to 6).

### G2-Hpulses and TMH

**Figure 2:**
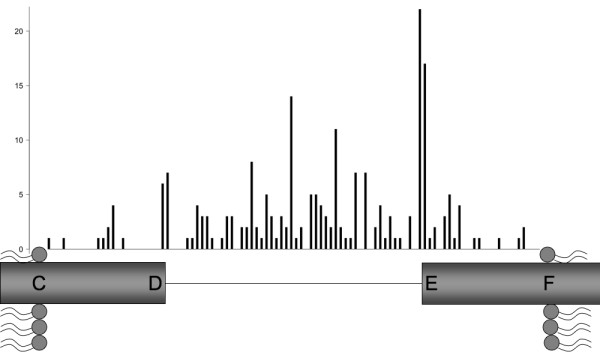
**G2-HPulse distribution**. This figure displays schematically the relative position of G2-HPulses within one of the three intervals: [C;D] is the extracellular end of the first TMH, [E;F] is the extracellular beginning of the next TMH and [D;E] comprises the amino acids positioned between the two TMHs. The length of [C; D], [D; E] and [E; F] is proportional to the number of involved amino acids.

First we wanted to determine whether the formation of α-helix bundles that characterize the TM proteins of our dataset was linked to the G2-HPulse distribution in their sequence. Therefore, we searched the position of G2-Hpulses for successive TMH: from the middle of the first TMS (or [B; C], as defined in Figure 1) to the middle of the second TMS (or [F; G]). We only considered situation where the length of [C; D] and [E; F] were strictly positive and where the length of [D; E] was smaller than 40 amino acids (those parameters will be constant for the whole study if not mentioned otherwise). We found 228 successive helices that filled these criteria. Specifically, no G2-Hpulse was found in one case, 13 (5.7%) G2-Hpulses were located in TMS and 214 (93.9%) G2-Hpulses were located between C and F as expected. In 27 cases, where multiple G2-Hpulses were detected, only one signal was selected (29 G2-Hpulses were thus discarded). Figure 2 displays schematically the relative distribution of the 214 G2-Hpulses located between C and F. The length of [D; E] corresponded to 52.3% of all amino acids located between C and F and included 73.8% of the identified G2-Hpulses. [C; D] (the extracellular TMH end) corresponded to 23.5% of all amino acids between C and F and contained 7.9% of G2-Hpulses, whereas [E;F] (the extracellular beginning of the next TMH) contained 24.2% of all amino acids and 18.2% of G2-Hpulses. A G2-Hpulse hot spot was identified at position E and one amino acid before.

### G2-HPulses discriminate between TMH

A well-defined TMH is characterized by the amino acids involved in its secondary structure(s) and in the embedded region. Thus, we needed to know whether G2-HPulses were linked to the structure boundaries or to the embedded core. If G2-Hpulses can discriminate between TMH, then they should be preferentially located between α-helices and therefore show a different distribution pattern in TM (TMS +/- 40 amino acids) or non-TM contexts. Indeed, in a non-TM context, the distribution of G2-Hpulses strictly followed the distribution of the amino acids (P = 1). Conversely, in a TM context, this distribution was strongly associated with the presence of α-helices (P < 0.0001) (Table 1).

**Table 1:** G2-HPulses and amino acid distribution in TM and non-TM contexts

TM	Helix	G2 distribution	%	Amino acid distribution	%
Yes	Yes	234	35.2	15466	65.0
Yes	No	431	64.8	8346	35.0
No	Yes	35	20.7	1033	20.5
No	no	134	79.3	4015	79.5

**Figure 3:**
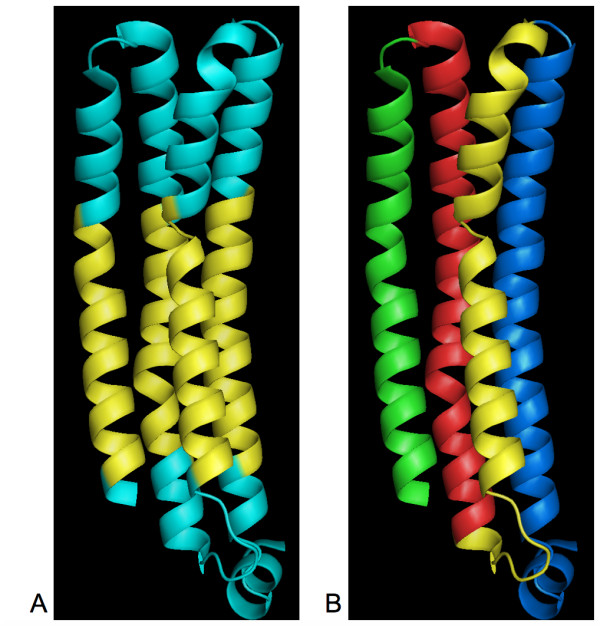
**Structure of the rotor ring of the V-type Na-ATPase**[15]. A: Embedded amino acids are in yellow (limits predicted by PDBTM). B: Each G2-HPulse is represented by a different color.

For example, the rotor ring of the V-type Na-ATPase [15] is composed of four long α-helices (32.5 amino acid-long on average) that extend well beyond the membrane (Figure 3A), and the four TMH are separated by 5, 6 and 5 amino acids respectively. As illustrated in Figure 3B, G2-HPulses predicted accurately the TMH extremities. Each TMH was identified as a single structural unit, even when it was composed of more than one α-helix. Each TMH began with a new G2-HPulse and the integrity of each TMH was conserved. The first and third G2-Hpulse were precisely located between two TMH, while the second one was positioned two amino acids after the start of the TMH.

### G2-HPulses and surrounding helices

Two TMHs can be separated by an interfacial helix; this is generally a short (less than 10 amino acids) α-helix that is often parallel to the membrane plane. We thus wondered whether the presence of an interfacial helix could be linked with the presence of a second G2-Hpulse. To answer this question, we compared the number of G2-Hpulses and the presence of α-helices in two successive TMHs. We found that the majority of TMHs that were not separated by an interfacial helix contained only one G2-Hpulse (Table 2); whereas the 13 TMHs with no helix and two G2-Hpulses were related to complex structures (Additional file 1). The majority of TMHs with one or more helices (74.4%) also had only one G2-Hpulse. These results indicate that while G2-Hpulses separate consecutive TMHs, they often associate surrounding helices with a TMH and thus interfacial helices are usually not predicted by G2-Hpulses.

**Table 2:** Number of G2-HPulses between two consecutive TMHs in relation to the presence of helices within this segment

**Number of G2-Hpulses**	**No helix between TMHs**	**One Helix between TMHs**	**More than one helix between TMHs**
0	1	0	0
1	161	32	6
2	13	11	3
> 2	1	0	0

The average length of α-helices between 2 TMHs was 8 AA.

### G2-Hpulses and the original Kyte-Doolittle algorithm

The algorithm of Kyte and Doolittle (KD) is used to calculate the distribution of hydrophobic segments in a sequence (thus predicting its 2D topology) and is based on a sliding window of 19 amino acids: if a position has an average value of hydrophobicity higher than the threshold of 1.6, it corresponds to a TMS. Unfortunately, many TMS do not show a hydrophobic peak as, despite a raise of hydrophobicity, the average value does not reach the threshold and thus are not identified by the KD algorithm. Therefore, we used our HPP tool to try to detect a G2-HPulse between the undetected TMS and the preceding TMS. Among the 129 TMS that did not contain a sufficiently high hydrophobic peak, 122 (94.6%) were correctly preceded by a G2-Hpulse.

### Association between secondary structures and G1-HPulses

**Figure 4:**
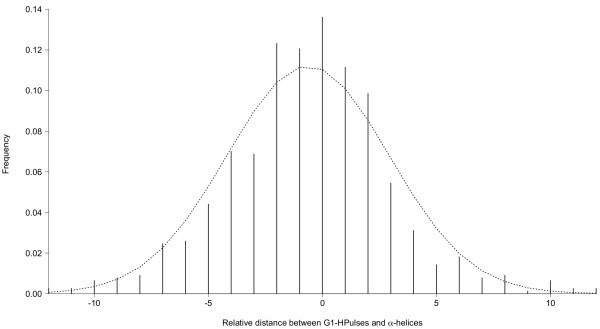
**Distribution of G1-HPulses compared to the extremities of α-helices**. A helix is considered to be in or near the membrane if its distance to the closest TMS is not higher than 40 amino acids: 782 helices were selected. Eleven values were not contained in the range [12] and thus were not displayed.

To test whether HPulses can influence the secondary structure of an IMP, we compared the G1-HPulse distribution with the α-helix extremities. To this aim, we wrote a program to automatically assign a unique G1-Hpulse to each extremity in order to locate the G1-HPulse that is closest to the beginning of an α-helix within or near the membrane (Figure 4). As the number of G1-Hpulses is greater than the number of helices, we can be sure that a G1-Hpulse is found for each extremity. This is somehow unsatisfying, because extremities associated with distant G1-Hpulses should be considered as 'not-detected'; however, we could not define a meaningful threshold for a maximum distance. Nevertheless, in the selected area (TMS +/- 40 amino acids), 1681 G1-HPulses were detected and 80.3% (628/782) of α-helices were associated with a G1-Hpulse, with a distance comprised in the range [-4; +4 amino acids].

### Structural irregularities and G1-HPulses

Structural irregularities of α-helices like re-entrant loops, kinks or partial helices may have a crucial functional role as illustrated by the potassium channel in which a partial helix mainly forms the selectivity filter and a tilted and slightly kinked helix forms the pore [16]. In order to assess the existence of a link between such structural irregularities and G1-HPulses, we focused on kinks, as they are a hallmark of TM proteins. We thus used the MC-HELAN method to detect kinks in α-helices of our dataset. We then compared the position of G1-Hpulses to five main structural events: begin/end of α-helices, begin/end of TMS and kinks. For all results, we decided to accept a 3 amino acid error.

This analysis showed that 63.1% (1061/1681) of G1-HPulses corresponded to these structural events. Among these G1-HPulses, 59.6% were related to α-helices extremities, 32.2% to TMS extremities and 8.2% to kinks. We then compared the position of kinks and that of G1-HPulses and of Prolines, which are the main kink inducers. With a 3 amino acid error, 104 (33.99%) Prolines and 129 (42.16%) G1-HPulses were found in the vicinity of the 306 reported kinks.

### Case studies

#### Lactose Permease

Channels are dynamic structures, so kinks may (dis)appear when moving from the open state to the closed state structure, but, unfortunately, very few of them have been crystallized in multiple states. One exception is represented by the lactose permease transporter whose crystal structure has been described in multiple conformations (Table 3) and for which five PDB files are available [17-19]. For each structure, after STRIDE analysis, we reported the beginning of each sub-helix and the G1-HPulse predictions (Table 4).

**Table 3:** Summary of the five available 3D structures of the lactose permease

PDB file	C154G mutant	Specificity	Resolution
1PV6	Yes	Native	3.5
1PV7	Yes	With bound substrate homolog	3.6
2V8N	No	Native	3.6
2CFP	Yes	Acidic pH (5.6)	3.3
2CFQ	Yes	Neutral pH (6.5)	2.95

The C154G lactose permease mutant, which binds to ligands but catalyzes little transport, is used to obtain well-diffracting crystals.

**Table 4:** Sub-helix extremities of lactose permease

2CFP	2CFQ	2V8N	1PV6	1PV7	G1-HPulses
-	-	-	2	2	-
**8**	**7**	**7**	**7**	**7**	**7**
30	29	-	-	-	27
**42**	**45**	**45**	**42**	**42**	**43**
59	63	60	-	-	61
-	75	75	74	74	74
87	86	-	-	-	82
-	95	94	-	-	89
**104**	**104**	**104**	**104**	**104**	**101**
**120**	**115**	**121**	**120**	**120**	**122**
143	-	-	140	140	136
-	149	147	-	-	153
-	166	166	166	166	166
177	179	-	-	-	174
-	-	-	-	-	*191*
**210**	**210**	**210**	**210**	**210**	**205**
**220**	**220**	**220**	**220**	**220**	**220**
235	-	232	-	-	-
244	-	243	-	-	242
**257**	**257**	**254**	**254**	**254**	**257**
269	-	265	-	-	268
**288**	**288**	**288**	**288**	**288**	**290**
**312**	**312**	**312**	**312**	**312**	**310**
325	-	325	-	-	323
-	343	346	346	346	341
357	-	358	357	357	359
**377**	**378**	**378**	**378**	**378**	**374**
-	-	-	-	-	*393*
-	-	-	408	408	404

For each TMH of each 3D model, we reported the start (amino acid position) of the sub-helices. G1-HPulse predictions are also reported. Positions found in all are bolded. Positions of G1-HPulses that were not found in any model are indicated in italic.

The position of the beginning of 10/27 sub-helices was the same in the five models and all 10 were associated with G1-HPulses, indicating that crucial structural positions are linked to G1-HPulses. Moreover, 92.6% (25/27) of α-helix starts were related to a G1-HPulse. Finally, whereas in the 1PV6 model of the lactose permease transporter 40.7% (11/27) of the G1-HPulses could be considered as false positives, this value dropped to 7.4% (2/27) when taking into account all conformations. This suggests that a G1-HPulse considered as a false positive prediction in one conformation may be a true positive prediction in another conformation. In addition, these signals could also correspond to other irregularities that were not detected by the MC-HELAN software.

**Figure 5:**
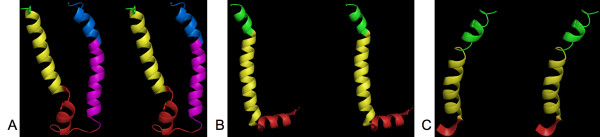
**Prediction of structural irregularities in the different 3D structures of the lactose permease by G1-HPulses**. Each G1-HPulse is indicated by a change of color. A, stereo view of the 2CFQ structure (from amino acid 5 to 71): the red α-helix is a partial helix and the transition from the magenta to the blue α-helix is marked by a kink. B, stereo view of the 1PV6 structure (from amino acid 209 to 250): the yellow α-helix is clearly isolated from the red and green α-helices. A kink separates the green and yellow α-helices. C, stereo view of 2V8N (from amino acid 311 to 343): the red and green α-helices form a curved α-helix whose interruption is detected by a G1-HPulse.

To test this hypothesis, we localized the G1-HPulses on the different 3D-structures of the lactose permease transporter. We noticed that G1-HPulses were not strictly associated with kinks, but rather with several types of irregularities. As illustrated in Figure 5, various irregularities could be associated with a G1-HPulses: kinks in Figures 5A and 5B, and α-helix interruptions in Figure 5A and 5C. We tested different G1-HPulses criteria (length, intensity, etc.) to discriminate between irregularities, but so far we have been unable to associate a specific G1-HPulse parameter with a type of structural irregularities (data not shown).

#### Chimeric voltage-dependent K^+ ^channel

**Figure 6:**
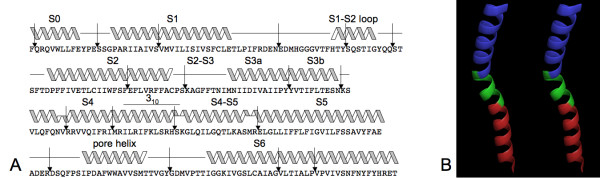
**Structure of the chimeric Kv channel**. A, schematic model of the structure, where arrows represent G1-HPulses (Figure adapted from Long *et al. *[20]). B, stereo view of S6: each G1-HPulse is indicated by a change of color.

Voltage-dependent K^+ ^(Kv) channels are found in neurons and muscle. A Kv chimera constructed from two Kv channels (Kv1.2 and Kv2.1) was selected for its particular structure and resolution (2.4 Å) [20]. As represented in Figure 6A, it is composed of 6 TMHs (from S1 to S6). HPulse predictions showed interesting correlations between G1-HPulses and structural events. Each interval separating two successive α-helices contained a unique G1-HPulse. In addition, each structural irregularity comprised between S3b and S5 was clearly detected (i.e., S3b, S4 irregularity, S4 3_10 _helix and S4-S5 helix). Two G1-HPulses within α-helices (i.e., S1 and S2) were not directly related with a reported irregularity and thus might not be relevant. On the other hand, other G1-HPulses within α-helices were strongly associated with structural irregularities as illustrated in Figure 6B, where the two G1-HPulses in S6 corresponded to apparent kinks.

## Discussion and Conclusions

In this study we defined two groups of hydrophobic pulses to predict the secondary structure of TM proteins: G2-HPulses for large structures, whose predictions were thus compared to the TMH boundaries, and G1-HPulses for small structural events, which were compared to the α-helix extremities.

**Figure 7:**
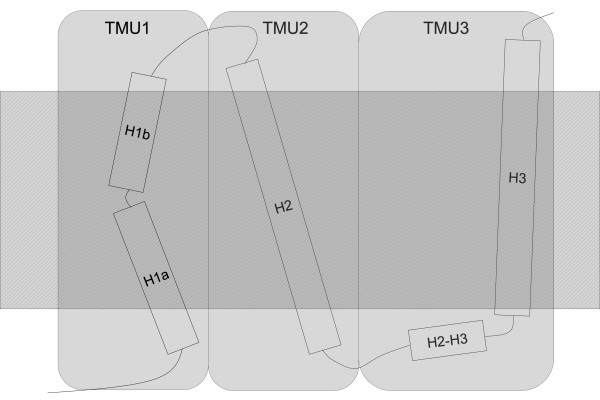
**The concept of transmembrane unit (TMU)**. The hatched area symbolizes the membrane. TMS correspond to the embedded part of α-helices. A TMH is composed of one (H2 and H3) or more (H1a and H1b) α-helices. The TMU groups together structures that are comprised between two G2-HPulses: it can contain a single α-helix, like TMU2, or associate TMH and small structures localized near the membrane, like TMU3 that contains H3 and H2-H3.

Hydrophobicity is normally used to detect TMS, but results obtained using G2-HPulses indicate that the structure of the whole TMH, and not only that of the embedded core, depends on hydrophobicity. As shown in Table 1, TMH were efficiently separated by G2-HPulses. Nevertheless, short helices (about 8 amino acids) were often associated with a TMH. As a consequence, a TM region could be regarded as a set of secondary structures, and G2-HPulses efficiently separated successive sets. Therefore, we propose the concept of transmembrane unit (TMU) (Figure 7), a structure that contains the TMS and is composed of one or more helices. Although a TMU can contain only one TMH, it can also include some small surrounding helices. During the process of IMP folding and insertion into the membrane, G2-HPulse distribution may illustrate the partitioning of the unfolded protein into the bilayer interface. This step may involve the TMU, which is a larger structure that embraces both TMH and TMS.

Furthermore, G1-HPulse distribution underlines another link between hydrophobicity and secondary structures. Indeed, the proximity of G1-HPulses and α-helix boundaries indicates that α-helices are strongly associated with HPulses. Altogether these data point to a new fundamental role for hydrophobicity: its variations are associated with secondary structures.

Channels are dynamic structures and as a result their topology is prone to change. The study of the different models of lactose permease showed that the majority of α-helix extremities identified in the five models are not used all at the same time, but are rather activated according to the channel state. Alternatively, some G1-HPulses could correspond to transient structures that are used during gating. We have no hypothesis on how G1-HPulses are selected in a structure, but the presence of very few α-helix extremities that are not linked to a G1-HPulse underlines the influence of hydrophobicity on the secondary structure of TMH.

Structural irregularities, such as kinks, have a function in some channels as they may serve as a point of flexure during gating. Their origin may be related to specific amino acid sequences; for instance, prolines are the main kink inducers and count for about a third of TMH kinks. Nevertheless, only 20% of all prolines cause a significant kink [21]: this implies that the presence of prolines cannot be considered as a stand-alone criterion. We found that more than 40% of kinks were related to a G1-HPulse. Moreover, as shown in Figure 5, G1-HPulses cannot distinguish the different structural irregularities and therefore the presence of G1-HPulses could be associated with other criteria.

As a conclusion, G1-HPulses suggested that variations of hydrophobicity in a small region defined a succession of weakness within the TM structures. Those weaknesses could correspond, depending on the context, to strat/end or irregularities in TMH.

Many bioinformatics tools have been developed and are available to predict the topology of embedded regions, thus biologists can already quite accurately localize TM segments. The Hydrophobic Pulse Predictor tool, described in this study, can be used to provide additional, new information on these regions. Indeed the HPP tool can predict from the primary amino acid sequence the global organization of a TM segment as G2-HPulses clearly distinguish the different TMU. In addition, G1-HPulses can pinpoint key changes in secondary structures within a TMU, even though some G1-HPulses are not related to an annotated structural event (36.9%). Nevertheless, as illustrated by the study of lactose permease, the number of false positives decreases with the availability of multiple conformations of a channel. Therefore, it is still difficult to assess the real number of false positive among G1-HPulses.

Overall, hydrophobic pulses seem to be a universal signal that is broadcasted along the peptide sequence and that is translated into structural events: α-helices (transient or not), irregularities or helix interruption. Although more studies on hydrophobic pulses are needed to fully understand their mechanics, these early results already indicate that hydrophobic pulses should be integrated in transmembrane proteins studies.

## Methods

### Hydrophobic pulse

A hydrophobic pulse is defined as a segment containing a raise followed by a decrease of hydrophobicity in a sequence. To detect pulses, we defined the score of hydrophobicity variation of one amino acid at position i in a sequence for sliding windows of different lengths. The score for a window of length n consisted of the difference between the sum of hydrophobicity (using the Kyte and Doolittle hydrophobicity scale [4]) of consecutive amino acids and the sum of hydrophobicity of the preceding amino acids, weighted by a sinus function (Equation 1).(1)

**Equation 1**. Hydrophobicity variation for a given amino acid (AA). AA(i) represents the amino acid at position i in the sequence and KD(x) represents the Kyte-Doolittle score for amino acid x. The score(i) involved (2*n+1) residues.

**Figure 8:**
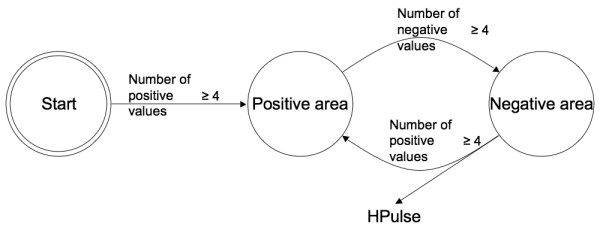
**Detection of HPulses by a finite state automaton**. The consensus is based on the sign of values that have been computed for 5 different lengths of window. The same automaton was used for both G1 and G2, but with different window sizes.

The value of n determines the size of the studied structural event. Since no HP associated with a single n value revealed all structural events, we could not use a single n value. Accordingly, we decided to create two groups of 5 values of n and established a consensus for each group. The G1 group (n = 2 to 6) focused on small TM structures, while G2 group (n = 12 to 16) focused on large TM For the first group (small structures), n = 2 represented the minimum amino acid sequence length for adopting an α-helix structure, which is characterized by hydrogen bonds linking amino acids at position i and i+4. An alpha-helix that is straight and perpendicular to the plane of the cell membrane needs about 21 residues to fit to the thickness of the membrane: this value corresponds to n = 10 and may represent the minimum length required to study transmembrane alpha-helices. Since two consecutive α-helices can be only one or two amino acids apart as reported for helix 4 and 5 of bacteriorhodopsin (PDB file 1C3W[22]), a window larger than 16 could contain two or more transmembrane α-helices. Therefore, we decided to exclude window sizes above n = 16 and limit the range for large TM structures to n = 12 to 16. Intermediate values (n = 7 to 11) were discarded, as they just represent intermediate states between G1 and G2. A finite state automaton created a consensus of variation for each group (Figure 8): the consensus was based on an agreement of at least 4 out of 5 values. A hydrophobic pulse was represented by the first position of each series of positive values: HPP computed hydrophobic pulses for both G1 and G2, which are called G1-HPulses and G2-HPulses.

### Benchmark

Our dataset contained 541 TMHs from 70 IMPs. We selected polytopic α-helical IMPs from the database of the Stephen White Laboratory http://blanco.biomol.uci.edu/index.shtml. We removed sequences with close homology; between two close models, we preferentially selected the model with the best resolution. CD-HIT [23] showed no redundancy within our dataset, with a threshold of 40% sequence homology (Additional file 2).

### Setting up limits

We used STRIDE [24] to identify the extremities of the α-helices. This allowed us to standardize the definition of α-helix boundaries within our dataset. TMS were defined using PDBTM [25,26], a tool that directly predicts embedded regions from PDB structures. TMH were automatically annotated; every helix comprised in a TM constituted a TMH, which implies that a TMH can contain more than one α-helix. Therefore, each residue has one structural state ('helical' or 'not helical') and one membrane state ('in membrane' or 'not in membrane') in this study.

### 3_10 _helices

The majority of IMPs in our dataset also contained 3_10 _helices. We decided not to consider this kind of helix as a structural event on its own, as many α-helices begin or/and end with a 3_10 _helix, often containing 3 amino acids. Therefore, in this study, all helix extremities were only related to α-helices and 3_10 _helices were not considered.

## Abbreviations

TM: transmembrane; TMU: transmembrane unit; 3D: three-dimensional; HPulse: hydrophobic pulse; IMP: integral membrane protein; PDB: protein data bank; TMS: transmembrane segments; TMH: transmembrane helix; KD: Kyte and Doolittle.

## Authors' contributions

DP developed the program. DP and CB carried out the analysis. DP, CB and MC participated in drafting the manuscript. All authors read and approved the final manuscript.

## Supplementary Material

Additional file 1 **The 13 cases where TMHs are separated by two G2-Hpulses and no interfacial helix (not recognized as α-helix by STRIDE)**. Each G2-HPulse is represented by a different color. A, 1OTS. B, 3BEH. C, 2Z73. D, 2NWL. E, 2VPZ. F, 3K3F. G, 2B2F. H, 1XQF. I, 2WIT. J, 2ZY9. K, 3B4R. L, 2BHW. Click here for file

Additional file 2 **Dataset of transmembrane proteins**. For each PDB file, the chains used in the dataset are also given. Click here for file
