# Supplementary figures and images for: Hydrophobic pulses predict transmembrane helix irregularities and channel transmembrane units
Source: BMC Bioinformatics. 2011 May 6;12:135. doi: 10.1186/1471-2105-12-135 (PMC3110554; doi:10.1186/1471-2105-12-135)

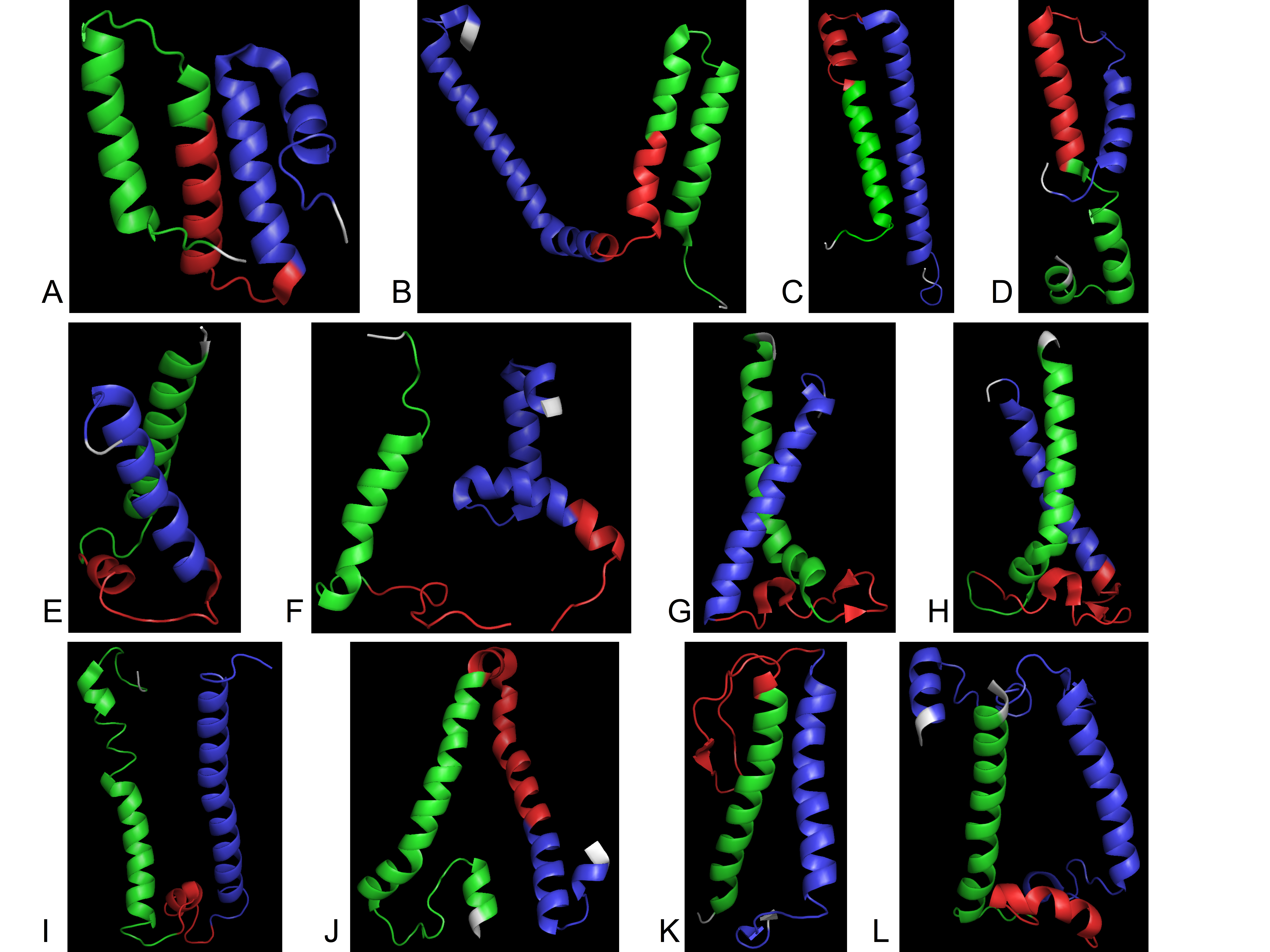

Supplement: Additional file 1 — The 13 cases where TMHs are separated by two G2-Hpulses and no interfacial helix (not recognized as α-helix by STRIDE). Each G2-HPulse is represented by a different color. A, 1OTS. B, 3BEH. C, 2Z73. D, 2NWL. E, 2VPZ. F, 3K3F. G, 2B2F. H, 1XQF. I, 2WIT. J, 2ZY9. K, 3B4R. L, 2BHW. [file 1471-2105-12-135-S1.TIFF]
