# Supplementary material for: Hydrophobic pulses predict transmembrane helix irregularities and channel transmembrane units
Source: BMC Bioinformatics. 2011 May 6;12:135. doi: 10.1186/1471-2105-12-135 (PMC3110554; doi:10.1186/1471-2105-12-135)
Supplement: Additional file 2 — Dataset of transmembrane proteins. For each PDB file, the chains used in the dataset are also given. [file 1471-2105-12-135-S2.DOC]

Supplementary Table 1: List of the 70 models used in this study (PDB code_chain).

1M0K_A, 1E12_A, 1XIO_A, 1H68_A, 3DDL_A, 1U19_A,

2Z73_A, 1K4C_C, 2AHY_A, 1LNQ_A, 1ORQ_C, 3JYC_A,

2A79_B, 3BEH_A, 2VL0_A, 3EAM_A, 2VV5_A, 2IUB_A,

2ZY9_A, 1YMG_A, 3KCU_A, 3K3F_A, 1XQF_A, 2B2F_A,

3B9W_A, 3B45_A, 2NR9_A, 3B4R_A, 1OTS_A, 2DHH_A,

2H8A_A, 2UUH_A, 1PW4_A, 3DH4_A, 2JLN_A, 2WIT_A,

3HQK_A, 3GIA_A, 2A65_A, 2NWL_A, 3H90_A, 1ZCD_A,

1OKC_A, 2QI9_A, 2HYD_A, 3D31_C, 2NQ2_A, 2R6G_F,

2R6G_G, 1P49_A, 1YEW_A, 1YEW_B, 1YEW_C, 2J8C_L,

2J8C_M, 2BHW_A, 1JB0_A, 3BZ1_A, 3BZ1_B, 3BZ1_C,

3BZ1_D, 1WPG_A, 2ZXE_A, 2BL2_A, 2WIE_A, 1L0V_C,

1L0V_D, 2BS2_C, 1KQF_C, 1NEK_C, 1NEK_D, 2FBW_C,

2BFW_D, 1Q16_C, 2VPZ_C, 1V54_A, 1V54_C, 1EHK_A,

1PPJ_C, 1VF5_A, 1Q90_D.
